# Inactivation of the Ecs ABC Transporter of *Staphylococcus aureus* Attenuates Virulence by Altering Composition and Function of Bacterial Wall

**DOI:** 10.1371/journal.pone.0014209

**Published:** 2010-12-02

**Authors:** Ing-Marie Jonsson, Jarmo T. Juuti, Patrice François, Rana AlMajidi, Milla Pietiäinen, Myriam Girard, Catharina Lindholm, Manfred J. Saller, Arnold J. M. Driessen, Pentti Kuusela, Maria Bokarewa, Jacques Schrenzel, Vesa P. Kontinen

**Affiliations:** 1 Antimicrobial Resistance Unit, Department of Infectious Disease Surveillance and Control, National Institute for Health and Welfare (THL), Helsinki, Finland; 2 Department of Rheumatology and Inflammation Research, University of Gothenburg, Göteborg, Sweden; 3 Genomic Research Laboratory, Service of Infectious Diseases, University Hospitals of Geneva, University of Geneva, Geneva, Switzerland; 4 Molecular Microbiology, Groningen Biomolecular Sciences and Biotechnology Institute, Kluyver Centre for the Genomics of Industrial Fermentations and the Zernike Institute of Advanced Materials, University of Groningen, Haren, The Netherlands; 5 Department of Bacteriology and Immunology, Haartman Institute, University of Helsinki, Helsinki, Finland; 6 Division of Clinical Microbiology, Helsinki University Central Hospital Laboratory, Helsinki, Finland; Institut de Pharmacologie et de Biologie Structurale, France

## Abstract

**Background:**

Ecs is an ATP-binding cassette (ABC) transporter present in aerobic and facultative anaerobic Gram-positive Firmicutes. Inactivation of *Bacillus subtilis* Ecs causes pleiotropic changes in the bacterial phenotype including inhibition of intramembrane proteolysis. The molecule(s) transported by Ecs is (are) still unknown.

**Methodology/Principal Findings:**

In this study we mutated the *ecsAB* operon in two *Staphylococcus aureus* strains, Newman and LS-1. Phenotypic and functional characterization of these Ecs deficient mutants revealed a defect in growth, increased autolysis and lysostaphin sensitivity, altered composition of cell wall proteins including the precursor form of staphylokinase and an altered bacterial surface texture. DNA microarray analysis indicated that the Ecs deficiency changed expression of the virulence factor regulator protein Rot accompanied by differential expression of membrane transport proteins, particularly ABC transporters and phosphate-specific transport systems, protein A, adhesins and capsular polysaccharide biosynthesis proteins. Virulence of the *ecs* mutants was studied in a mouse model of hematogenous *S. aureus* infection. Mice inoculated with the *ecs* mutant strains developed markedly milder infections than those inoculated with the wild-type strains and had consequently lower mortality, less weight loss, milder arthritis and decreased persistence of staphylococci in the kidneys. The *ecs* mutants had higher susceptibility to ribosomal antibiotics and plant alkaloids chelerythrine and sanguinarine.

**Conclusions/Significance:**

Our results show that Ecs is essential for staphylococcal virulence and antimicrobial resistance probably since the transport function of Ecs is essential for the normal structure and function of the cell wall. Thus targeting Ecs may be a new approach in combating staphylococcal infection.

## Introduction

The increased bacterial resistance to most or even all current antibiotics is a threat that necessitates development of new molecules with novel targets and/or modes of action [1]. Cell components which are dispensable for growth but indispensable for virulence and pathogenesis constitute therefore an interesting class of potential candidates. In particular, bacterial membrane proteins, including transporters, represent a wide repertoire of likely antimicrobial targets for developing such new therapies. Bacterial ATP-binding cassette (ABC) transporters are involved in the uptake or secretion of a large variety of different biomolecules, nutrients, antimicrobial agents or ions across the cytoplasmic membrane. Some of these transporters are involved in virulence and may represent appropriate antimicrobial targets [2] as exemplified by the iron uptake systems [3]–[5]. The basic structure of ABC transporters, independently of whether they are exporters or importers, consists of a transmembrane permease domain and a cytoplasmic ATP-binding domain associated with the permease. Both, the permease domain and the ATP-binding domain are homo- or heterodimers, and depending on the transporter, encoded by 1 to 4 different genes. Additionally, nearly all bacterial importers also have a periplasmic high-affinity solute-binding component. The ATP-binding domain hydrolyzes ATP and energizes the molecular transport.

Ecs is an as yet poorly characterized ABC transporter. It is present in aerobic and facultative anaerobic Gram-positive Firmicutes, while no Ecs has been detected with amino acid sequence homology surveys from Actinobacteria, obligatory Gram-positive anaerobes or Gram-negative bacteria. Ecs was originally identified in *Bacillus subtilis* in a mutant screen for decreased secretion of overexpressed α-amylase [6], [7]. Characterization of *B. subtilis ecs* mutants showed that secretion of overexpressed extracellular proteins was reduced and signal peptides were inefficiently processed in the mutants due to a defect in the late stage of secretion [8], [9]. The defect in signal peptide processing was partially suppressed by overexpressing a type I signal peptidase [9]. In addition to the secretion defect, *ecs* mutants are defective in transformation competence [7] and biofilm formation [10]. Recently it was shown that Ecs influences intramembrane proteolysis through the RasP protease [11]. On the other hand, inactivation of RasP causes a protein secretion defect similar to that of *ecs* mutant [11], suggesting that the impaired secretion is a result of the inhibition of intramembrane proteolysis. The *ecsA* and *ecsB* genes encode the homodimeric ATP-binding and permease domains of Ecs, respectively. It is still unknown which molecule(s) Ecs transport(s) and whether it functions as an exporter or an importer. The inhibition of intramembrane proteolysis suggests that Ecs may have a “cleaning function” in the membrane, e.g. it could remove inhibitors of RasP such as peptides from the membrane either into the cytoplasm or the extracellular medium.

*Staphylococcus aureus* strains resistant to multiple antibiotics are increasingly common causes of serious and problematic infections both in hospitals and communities [12], [13]. The pleiotropic phenotype of *B. subtilis ecs* mutants suggests that Ecs could be important for virulence in Gram-positive pathogens such as *S. aureus*. We constructed two null mutations of *S. aureus ecsAB* and studied their effects on virulence in a murine arthritis model [14], performed a DNA microarray analysis to assess the effects of an *ecs* mutation on global gene expression, determined antimicrobial susceptibilities of an *ecs* mutant and characterized the mutant phenotype also more generally. The mutant phenotype clarifies the functional role of Ecs in *S. aureus* and other gram-positive bacteria as well as the potentiality as a novel target for antimicrobial drug development.

## Results

### Construction of the *ecsAB* mutant strains

The *S. aureus ecsAB* operon (ORFs NWMN_1728 and NWMN_1727 in the Newman strain, respectively) was identified with sequence similarity of the deduced amino acid sequences of EcsA and EcsB proteins with the corresponding Ecs proteins of *B. subtilis*, as well as with the conserved localization of the operon in the genome close to the gene (NWMN_1729) encoding a Hit-family protein. In order to study the functional role of the Ecs ABC transporter in *S. aureus*, we constructed two null mutations of the *ecsAB* operon, one which deleted large parts of both *ecsA* and *ecsB* genes (Δ*ecsAB*) and another one which inserted about a 0.9 kb TargeTron intron fragment into *ecsA* (*ecsA*::intron) (see Materials and Methods). The Δ*ecsAB* (RH7636) and *ecsA*::intron (RH7783) mutations are in the *S. aureus* LS-1 [14] and Newman [15] strains respectively. We used these two mutants to study phenotypic effects of Ecs inactivation. We also constructed the pKTH3832 plasmid which carries the *ecsAB* operon in the pEPSA5 vector under transcriptional control of the xylose-inducible T5X promoter [16]. pKTH3832 was used to complement *ecs* mutation and verify the Ecs-dependency of some of the observed phenotypes.

### Growth, autolysis and lysostaphin sensitivity of *S. aureus ecs* mutant

**Figure 1:**
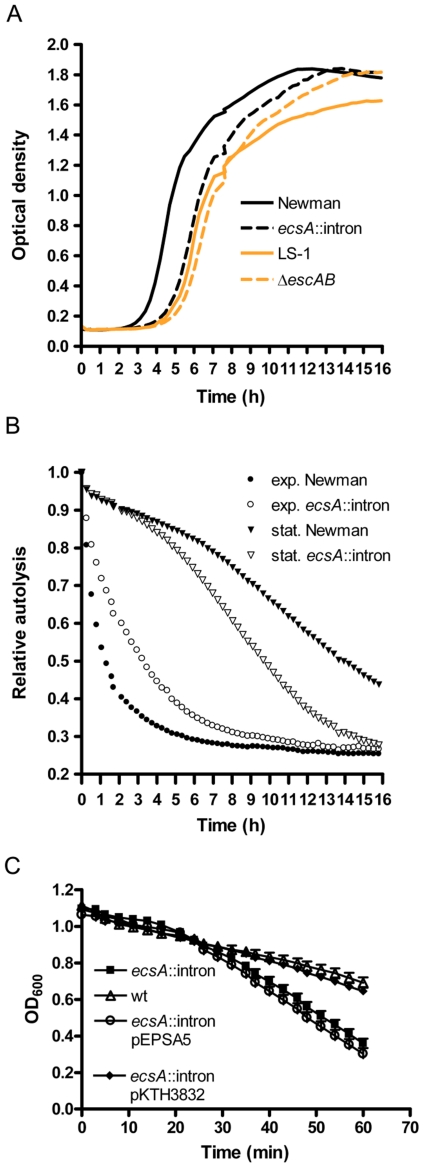
Growth, autolysis and lysostaphin sensitivity of *S. aureus ecs* mutant. (A) Growth curves of *S. aureus ecs* mutants and their wild-type parental strains in BHI growth medium as measured with a Bioscreen C apparatus. Lines are averages of five overlapping replicates of a single experiment. Repetition in standardized conditions gives the same result. (B) Autolysis of cells over time. Circles mark *S. aureus* cells harvested from the exponential growth phase (Klett 200–250) and triangles cells harvested from stationary phase cultures (Klett 700–720). Data points are averages of nine overlapping replicates of the same experiment. Samples from young cultures produced hyperbolic autolysis curves with gradual growth phase-dependent procession through shouldered curves to bent slopes of stationary phase cultures shown here with triangles. Baselines recorded with lysis buffer were in the range of 0.20 to 0.23 for these normalized curves. (C) Decrease in optical density over time of cell suspensions of the *ecsA*::intron mutant and the wild-type Newman strain treated with 10 µg ml^−1^ lysostaphin. Means of five determinations and error bars are shown.

Cultivation of the *ecs* mutants in BHI medium with a Bioscreen apparatus suggested that Ecs deficiency has a slight effect on growth. A difference in onset of growth was seen repeatedly with *ecsA*::intron mutant and wild-type Newman strain (Figure 1A). Otherwise the mutant grew in a similar manner as the wild-type strain throughout the exponential growth phase. The LS-1 strain was observed to grow more slowly and produce lower final optical densities as compared to the Newman strain in the same growth conditions (Figure 1A). However, the Δ*ecsAB* mutant grew in a similar manner as the *ecsA*::intron mutant exhibiting no reduction in the stationary phase optical density and only a slight delay in the growth onset as compared to the parental LS-1 strain.

We also examined whether the absence of Ecs affects autolysis and sensitivity to lysostaphin (see Materials and Methods for the assays). Autolytic activity of the cultures was found to be dependent on the growth phase from which cells were harvested for the assay (Figure 1B). Variation of results with actively growing cells was significant and, if anything, it was observed that Ecs deficiency slightly inhibited autolysis of cells during the exponential phase. In contrast, it was consistently seen that the absence of Ecs increased autolytic activity of mature stationary-phase cells. The *ecsA*::intron mutant is also more sensitive to lysostaphin (Figure 1C). Both phenotypes were complemented by pKTH3832, indicating that they are caused by the Ecs deficiency (the complementation of the lysostaphin sensitivity is shown in Figure 1C). These results suggest that Ecs transporter is important for the normal structure and/or function of the cell wall.

### Levels of cell wall proteins are decreased in the *ecsA*::intron mutant

**Figure 2:**
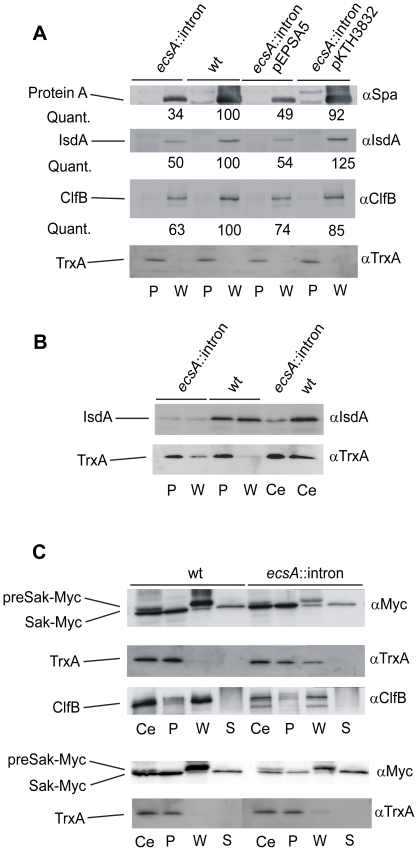
Effect of Ecs deficiency on exported proteins. Levels of three chromosomally-encoded cell wall proteins (Protein A, IsdA and ClfB) and plasmid-encoded secretory protein (Myc-tagged staphylokinase) in the *ecsA*::intron mutant and the parental Newman strain were determined by immunoblotting. (A) The protein levels in protoplasts (P) and protoplast supernatants containing the cell wall material (W) are shown. The expression of P*xyl*-*ecsAB* in the pKTH3832 plasmid was induced with 0.02% xylose. The protein levels were quantitated by determining optical densities of the protein bands and normalizing them to the TrxA protein in the protoplasts. The normalized protein amounts were adjusted so that the level in the wild-type Newman strain is 100. (B) Levels of IsdA and TrxA in whole cells (Ce) of the *ecsA*::intron mutant and the wild-type strain grown in iron-depleted BHI medium, and in their protoplast and wall fractions. (C) Immunoblot analysis of preSak-Myc and Sak-Myc levels in whole cells, protoplasts, cell wall and culture supernatant (S). The expression of P*xyl-sak-myc* in the pKTH3834 plasmid was induced with 0.5% xyloxe (upper panels) or 0.02% xylose (lower panels). TrxA and ClfB levels in the same samples (upper panels) were determined to control stability of the protoplasts and fractionation of the cell wall, respectively. The supernatant samples correspond to 8 µl, while the other samples are from 80 µl of the cultures.

The cell wall defect of *ecs* mutant suggested by the above results may also be manifested as changed levels of cell wall-associated proteins. Therefore, we determined amounts of three cell wall proteins in the *ecsA*::intron mutant and the wild-type strain by immunoblotting. The determined proteins are Protein A (Spa), IsdA iron-regulated heme-iron binding protein and ClfB clumping factor B precursor. Protein A could be detected with specific monoclonal mouse antibodies raised against Protein A. It was found that *ecsA*::intron mutation clearly decreased Protein A levels in the wall (about 3-fold as compared to the wild-type strain; Figure 2A). Transcriptome data (see below) showed that this decrease is at least partly caused by a decrease in *spa* gene transcription. The levels of IsdA and ClfB proteins were also lower in the wall of the mutant (grown in iron-sufficient BHI medium), but the difference was less marked (Figure 2A). pKTH3832 restored wild-type levels of the three proteins, indicating that their decrease was dependent on Ecs. Furthermore, since the expression and subcellular distribution of IsdA is regulated by iron availability [17], we also studied the effect of *ecsA*::intron on IsdA level in iron-depleted BHI medium. The *ecs* mutant grew slower than the wild-type Newman strain in this modified medium (data not shown), but we could prepare whole cell, lysostaphin protoplast and cell wall samples from the cells at the culture density of Klett 60 and perform the analysis. Immunoblotting showed that IsdA levels were clearly lower in all these samples as compared to the corresponding ones of the wild-type strain (Figure 2B). These results indicate that levels of at least some cell wall proteins are dependent on Ecs and further corroborate the functional role of Ecs in the cell wall.

### EcsAB-dependent accumulation of overexpressed staphylokinase precursor in the cell wall

In *B. subtilis*, Ecs inactivation impairs signal peptide processing and secretion of overexpressed secretory proteins. This phenotype is particularly clear with the heterologous *Bacillus amyloliquefaciens* AmyQ α-amylase [8], [9]. We wanted to find out whether absence of EcsAB causes a similar protein secretion defect in *S. aureus*. As a model secretory protein we used staphylokinase modified with C-terminal Myc-tag (Sak-Myc). The Sak-Myc protein was overexpressed from pKTH3834, a derivative of pEPSA5 carrying the *sak*-*myc* gene under the control of P_T5X_, by inducing with 0.5% xylose. The secretion and processing of Sak-Myc was also studied at a lower expression level induced with 0.02% xylose. The expression was induced at the cell density of Klett 70 and samples for immunoblotting were taken at Klett 200. The wild-type strain grew from Klett 70 to Klett 200 in one hour and the *ecsA*::intron mutant in two hours, indicating that overexpression of a secretory protein causes severe stress and growth inhibition in the mutant. Staphylokinase levels in whole cells, lysostaphin protoplasts, protoplast supernatant and culture supernatant were analyzed by immunoblotting with anti-Myc antibodies.

In both wild-type strain and *ecsA*::intron mutant, two forms of Myc-tagged staphylokinase, the preSak-Myc precursor and mature Sak-Myc, were detected (Figure 2C). The level of mature Sak-Myc was similar in these strains or even slightly higher in the mutant (whole cells and protoplasts), but the precursor level was lower in the mutant (whole cells). In protoplast supernatants containing the cell wall material, a clear difference was observed between the two strains. Surprisingly, in the wild-type strain, an abundant amount of preSak-Myc was accumulated in the wall, whereas none was detected in the protoplasts, suggesting that the precursor was almost totally located in the cell wall. In contrast, only a very low amount of preSak-Myc was detected in the corresponding cell wall fraction of the mutant and no precursor was observed in the protoplasts (Figure 2C). The *ecs* mutation also decreased slightly the level of secreted mature Sak-Myc in the culture supernatant. The same preparations were also immunoblotted with antibodies recognizing thioredoxin (TrxA) [18], a cytoplasmic protein, to control protoplast stability, and ClfB [19], to control fractionation of the subcellular fractions. In the case of wild-type strain, TrxA was detected in the whole cell and protoplast fractions in similar amounts but not in the wall fraction, indicating that these protoplasts were stable. There was some leakage of TrxA from the protoplasts of the mutant suggesting that these cells are less stable. We did not see any degradation band of preSak-Myc in the samples implying that increased proteolytic degradation in the mutant does not cause the difference in the preSak-Myc levels in the walls. These results suggest that EcsAB may be directly or indirectly involved in the release of preSak from the membrane into the wall. The immunoblotting of ClfB suggested that staphylokinase overexpression enhanced the effect of *ecs* mutation on ClfB level in the wall (Figure 2). There is probably competition in the export pathway between preSak-Myc and ClfB, and the export is somehow dependent on Ecs.

When P*xyl-sak-myc* was expressed at a lower level (induction with 0.02% xylose), the pattern was similar, but the amounts of the staphylokinase forms in whole cell, protoplast and cell wall fractions of the mutant were lower than in the corresponding fractions of the wild-type strain (Figure 2C). There was no difference in the level of the secreted Sak-Myc.

Ecs might affect indirectly the export of cell wall proteins and overexpressed preSak-Myc by modulating proton motive force (pmf). Therefore, we studied whether *ecsA*::intron mutation altered pmf. The *ecsA*::intron mutant and wild-type Newman strain showed a similar pmf as monitored with the fluorescent dye diSC_3_(5) (data not shown), indicating that the pleitropic phenotype of the mutant is not an indirect effect of altered pmf.

### Gene expression changes conferred by the *ecsA*::intron mutation

The pleiotropic phenotype of the *ecs* null mutant suggests that an Ecs defect causes major changes in global gene expression. We used DNA microarray to analyze Ecs-dependent changes in the transcriptome. The analysis was performed with the *ecsA*::intron mutant and the wild-type *S. aureus* Newman strain at two different growth phases: late exponential (3 h) and early stationary (6 h) phases.

At medium stringency of the analysis around 7% (3 h) and 10% (6 h) of the transcriptomes were differentially expressed in the *ecsA*::intron mutant as compared to the wild-type strain. The growth time had a significant impact on the transcriptome; 30–35% of the differentially-expressed genes were present in both lists (Figure S1, Table S1 and Table S2). A prominent feature of the transcriptome was the induction or repression of numerous genes (n = 64) encoding transporter proteins, particularly ABC-type transporters and PTS systems (Table 1). The fold changes of transporter gene expression were mostly around 2, but several higher differences were also observed. In particular, the *lacF* gene, which encodes a PTS system lactose-specific IIA component, was strongly repressed in the *ecsA*::intron mutant with almost a 20- and 40-fold difference at 3 h and 6 h, respectively. Since *ecsA*::intron is an insertion at the position 309/310 in the 738 bp *ecsA* gene, the oligonucleotide array used in the analysis detected the truncated *ecsA*' mRNA (MWNM_1728), but its level was reduced compared to the wild-type *ecsA* mRNA. The expression change of 36 genes (excluding *ecsAB*) was more than 4-fold at least in either of the two growth phases (Table 2). Most of these strongly differentially expressed genes were induced in the *ecsA*::intron mutant; only eight were repressed. Interesting, NMWN_0903 encoding a functionally unknown ABC transporter was repressed in the late exponential phase but strongly induced in the early stationary phase. The whole capsular polysaccharide biosynthesis operon *cap* was induced 2 to 5 fold in the mutant (Table 2, Table S1 and Table S2). The Ecs defect also affected the expression of genes involved in the biosynthesis of the cell wall and ribosomes, and numerous genes with a role in virulence. The NWMN_1655 gene, which encodes the virulence factor regulator protein Rot, was expressed at decreased levels (about 3-fold) in the *ecsA*::intron mutant at both growth phases. Since Rot is a positive regulator of *spa* gene expression [20], the down-regulation may explain why *spa* was expressed at strongly decreased levels (17-25-fold) in the mutant. The decreased *spa* expression is consistent with the decreased Protein A level in the wall of the mutant (see above). The *sdrC* and *sdrD* genes, which are required for adherence of *S. aureus* Newman to squamous epithelium in the anterior nares [19], were repressed in the null mutant. Furthermore, several genes encoding Spl serine proteases, superantigen-like proteins, accessory regulators, SsaA secretory antigens, staphylokinase, fibrinogen-binding proteins and a fibronectin-binding protein were differentially expressed (Table S1 and Table S2).

**Table 1:** Transporter systems differentially expressed in the *ecsA*::intron mutant.

Gene	Transporter protein	Fold change (wt/*ecsA*)	
		3 h	6 h
*bsaF*	ABC transporter		1.8
*cbiO*	cobalt transporter ATP-binding subunit		0.5
*fhuG*	ferrichrome ABC transporter (permease)		0.6
*glcA*	PTS system glucose-specific component	2.6	
*lacF*	PTS system lactose-specific IIA component	18.0	42.0
*mnhD*	monovalent cation/H^+^ antiporter subunit D	1.9	
NWMN_0048	drug transporter		2.0
NWMN_0136	PTS system component	2.7	
NWMN_0153	maltose ABC transporter permease protein	2.7	
NWMN_0154	maltose ABC transporter permease protein	2.5	
NWMN_0199	PTS system IIA component		0.5
NWMN_0211	ABC transporter ATP-binding protein		1.9
NWMN_0343	ABC transporter ATP-binding protein	0.5	
NWMN_0601	ABC transporter substrate-binding protein	1.8	
NWMN_0602	iron (chelated) ABC transporter permease protein	1.8	
NWMN_0688	ABC transporter ATP-binding protein		0.5
NWMN_0690	ABC transporter		1.9
NWMN_0691	amino acid ABC transporter permease protein		1.9
NWMN_0696	di-/tripeptide ABC transporter		1.9
NWMN_0861	oligopeptide ABC transporter substrate-binding protein	0.4	
NWMN_0863	oligopeptide ABC transporter ATP-binding protein	0.2	
NWMN_0903	ABC transporter ATP-binding protein	2.3	0.1
NWMN_0943	cobalt transport		2.2
NWMN_0968	spermidine/putrescine ABC transporter binding protein		1.8
NWMN_1231	ABC transporter ATP-binding protein	0.4	0.5
NWMN_1261	glycine betaine transporter		1.8
NWMN_1291	peptide ABC transporter ATP-binding protein	0.6	
NWMN_1292	oligopeptide transporter permease	0.5	
NWMN_1293	oligopeptide transporter permease	0.6	
NWMN_1458	ABC transporter permease		0.5
NWMN_1540	preprotein translocase YajC subunit	2.7	
NWMN_1728	ABC transporter ATP-binding protein	2.2	7.0
NWMN_1749	amino acid ABC transporter ATP-binding protein		3.1
NWMN_1763	ABC transporter ATP-binding protein		2.1
NWMN_1867	ABC transporter ATP-binding protein		1.8
NWMN_1950	ammonium transporter		0.4
NWMN_2050	cation efflux	0.5	0.5
NWMN_2076	FecCD iron compound ABC transporter permease		0.5
NWMN_2081	transporter		0.2
NWMN_2089	BCCT family osmoprotectant transporter		2.1
NWMN_2224	PTS system component	2.1	1.8
NWMN_2246	sodium/glutamate symporter	2.5	2.4
NWMN_2261	ABC transporter ATP-binding protein HtrB		2.2
NWMN_2268	L-lactate permease	3.0	
NWMN_2279	PTS system sucrose-specific IIBC component	2.4	
NWMN_2311	amino acid ABC transporter ATP-binding protein		2.0
NWMN_2343	drug transporter	0.5	
NWMN_2349	amino acid permease		0.6
NWMN_2370	transporter	0.5	0.5
NWMN_2412	ABC transporter ATP-binding protein		1.9
NWMN_2413	ABC transporter permease	4.5	
NWMN_2458	cation transporter E1-E2 family ATPase	1.8	2.6
NWMN_2521	permease domain-containing protein	2.0	
NWMN_2540	PTS system fructose-specific IIABC component		0.5
NWMN_2592	sodium sulfate symporter		1.8
NWMN_2593	RarD protein		3.5
*opp1F*	peptide ABC transporter ATP-binding protein	2.2	
*opuCD*	amino acid ABC transporter permease protein		2.0
*pstB*	phosphate ABC transporter ATP-binding protein	0.6	2.1
*ptsG*	PTS system glucose-specific IIABC component	1.9	0.3
*sirB*	ABC transporter permease protein SirB	1.8	
*tatA*	mttA/Hcf106 -related protein	0.5	
*uhpT*	sugar phosphate antiporter	2.6	
*ulaA*	ascorbate-specific PTS system enzyme IIC	1.8	2.7

**Table 2:** Genes induced or repressed more than 4-fold in the *ecsA*::intron mutant.

Gene	Protein	Fold change(wt/*ecsA*)	
		3 h	6 h
*capB*	capsular polysaccharide biosynthesis protein CapB	0.2	0.5
*capC*	capsular polysaccharide biosynthesis protein CapC	0.2	0.4
*capG*	capsular polysaccharide biosynthesis protein CapG	0.3	0.2
*capI*	capsular polysaccharide biosynthesis protein CapI	0.3	0.2
*fbp*	fructose-1,6-bisphosphatase		4.4
*gapB*	glyceraldehyde 3-phosphate dehydrogenase 2	4.9	
*hisD*	histidinol dehydrogenase	0.2	0.3
*hisZ*	ATP phosphoribosyltransferase regulatory subunit	0.0	0.2
*lacF*	PTS system lactose-specific IIA component	18.0	42.0
*lukE*	leukotoxin LukE	0.1	
NWMN_0074	glycosyl transferase group 1		0.2
NWMN_0353	ParB family chromosome partioning protein	4.1	
NWMN_0542	VraX (SAS016)		0.2
NWMN_0863	oligopeptide ABC transporter ATP-binding protein	0.2	
NWMN_0903	ABC transporter ATP-binding protein	2.3	0.1
NWMN_0995	bacteriophage L54a antirepressor	0.2	
NWMN_1223	hypothetical protein		0.1
NWMN_1252	hypothetical protein		0.2
NWMN_1445	hypothetical protein		0.2
NWMN_1623	transglycosylase domain-containing protein		4.6
NWMN_1690	hypothetical protein		4.6
NWMN_1728	ABC transporter ATP-binding protein EcsA	2.2	7.0
NWMN_1889	hypothetical protein		0.2
NWMN_1893	phage head-tail adaptor	0.1	
NWMN_2043	general stress protein 20U		0.2
NWMN_2081	hypothetical protein		0.2
NWMN_2199	secretory antigen SsaA	0.2	0.2
NWMN_2203	secretory antigen SsaA	0.2	0.2
NWMN_2468	acetyltransferase GNAT		0.2
*phoB*	alkaline phosphatase III	0.2	
*pnp*	purine nucleoside phosphorylase	5.3	
*pyrAA*	carbamoyl phosphate synthase small subunit		0.1
*pyrB*	aspartate carbamoyltransferase catalytic subunit		0.2
*pyrC*	dihydroorotase		0.2
*set3nm*	superantigen-like protein		0.2
*spa*	protein A	17.4	25.1
*trpE*	anthranilate synthase component I	0.1	0.1

### The effects of Ecs deficiency on the structure of bacterial surface

**Figure 3:**
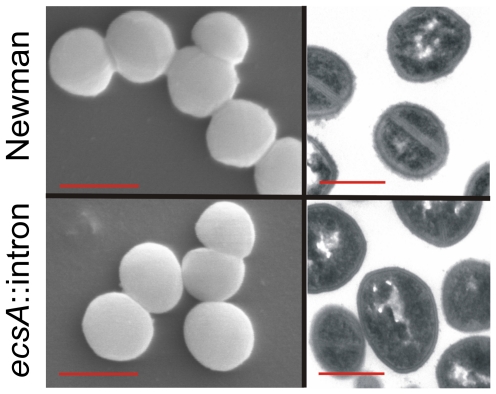
Effect of Ecs deficiency on cell surface texture. Scanning (left panels) and transmission (right panels) electron microscope images of *ecsA*::intron mutant and wild-type Newman cells. The scale bars 1 µm (left panels) and 0.5 µm (right panels). The scanning electron micrographs show smoother surface texture of the mutant as compared to the wild type but no significant difference in cell size. The transmission electron micrographs suggest that on the average cell surfaces of *ecsA*::intron mutant cells are more compact.

The induction of the capsular polysaccharide operon in *ecsA*::intron mutant (DNA microarray analysis above) suggested that the mutant might have a thicker capsule than the wild-type Newman strain. However, Indian ink-crystal violet staining of the capsule did not reveal increased amounts of capsule material in the mutant but rather suggested that *ecs* mutant cells are smaller than wild-type cells (data not shown). This was further studied by using electron microscopy, but the resulting images did not confirm the cell size difference. Instead, Ecs deficiency was found to alter cell surface. Wild-type Newman cells looked slightly fluffy by scanning electron microscopy, whereas *ecs* mutant cells were smoother (Figure 3). Accordingly, poorly defined, loose outer surface structure of the Newman strain and more sharply defined, compact surface structure of the *ecsA*::intron mutant were seen in transmission electron micrographs (Figure 3). Transformation of the mutant with pKTH3832 restored the wild type-like surface structure when P*xyl*-*ecsAB* was induced at a low level (not shown), indicating that the phenotype was complemented. The surface change of the mutant may explain why crystal violet-stained *ecs* mutant cells looked optically smaller than corresponding wild-type cells. The surface change is also consistent with the other results above (Figure 1A) showing that Ecs deficiency causes a growth defect as measured spectrophotometrically and has significant effects on the cell wall. Similarly, electron microscopy of LS-1 and the corresponding Δ*escAB* mutant strains revealed thicker cell wall and a minor change in surface structure which is consistent with the results in Figure 1A (data not shown).

### Ecs deficiency attenuated staphylococcal infection in mice

**Figure 4:**
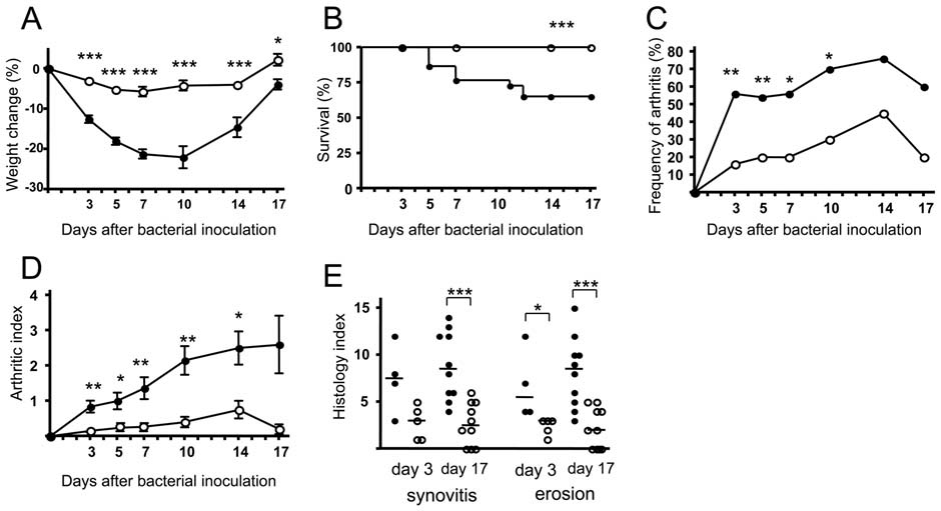
Weight change, mortality and arthritis during *in vivo S. aureus* infection in mice. (A) Weight change, (B) mortality, (C) frequency of arthritis, and (D) severity of clinically assessed arthritis in mice inoculated with the Δ*ecsAB* mutant or LS-1 wild-type *S. aureus* strain. (E) Histopathological evaluation of synovitis and joint erosion at day 3 and 17 after bacterial inoculation. Bars show means ± SEM. Horizonal lines indicate medians. Open circles Δ*ecsAB*, closed circles LS-1. *P<0.05; **P<0.01; ***P<0.001.

The importance of the *ecs* for *S. aureus* virulence during *in vivo* infection was studied in a mouse model of *S. aureus* arthritis [14]. Healthy mice were inoculated with *ecs* mutant or wild-type strain in four independent experiments (Table S3). Staphylococcal infection was followed by clinical signs as weight loss, mortality and frequency of arthritis. The results were consistent in all experiments and showed that mice infected with *ecs* mutant had significantly less weight loss, mortality and lower frequency and severity of clinically-assessed arthritis as compared with the mice inoculated with wild-type strain (Figure 4A–D). In addition, histological evaluation of joints obtained at days 3 and 17 revealed that the Δ*ecsAB* mutant caused milder synovitis and less bone erosions than the LS-1 strain (Fig. 4E). Inoculation of the *ecsA*::intron mutant and wild-type Newman strains supported the results obtained in the infection with Δ*ecsAB* mutant and wild-type LS-1 strains with respect to weight loss and frequency of arthritis (data not shown).

The ability of the *ecs* mutants to persist in the infected mice was studied by determining bacterial counts in kidneys obtained at days 3, 14 and 17 after bacterial inoculation (Table S3). Both *ecs* mutant strains had a significantly reduced capacity to persist in kidneys at the late phase of the infection (day 17), while the capacity at the early phase of the infection (day 3) was similar with the wild-type strains. The systemic inflammatory response induced by the *ecs* mutant and wild-type strains was assessed by the serum levels of IL-6. The Δ*ecsAB* mutant strain induced similar levels of IL-6 as the wild-type LS-1 strain, both on day 3 (pg/ml: 529 [400 to 1919] versus 773 [504–2750]) and on day 17 (pg/ml: 165 [100–312] versus 256 [150–344]).

### The Δ*ecsAB* mutant is protected from the bactericidal effect of α-defensins

**Figure 5:**
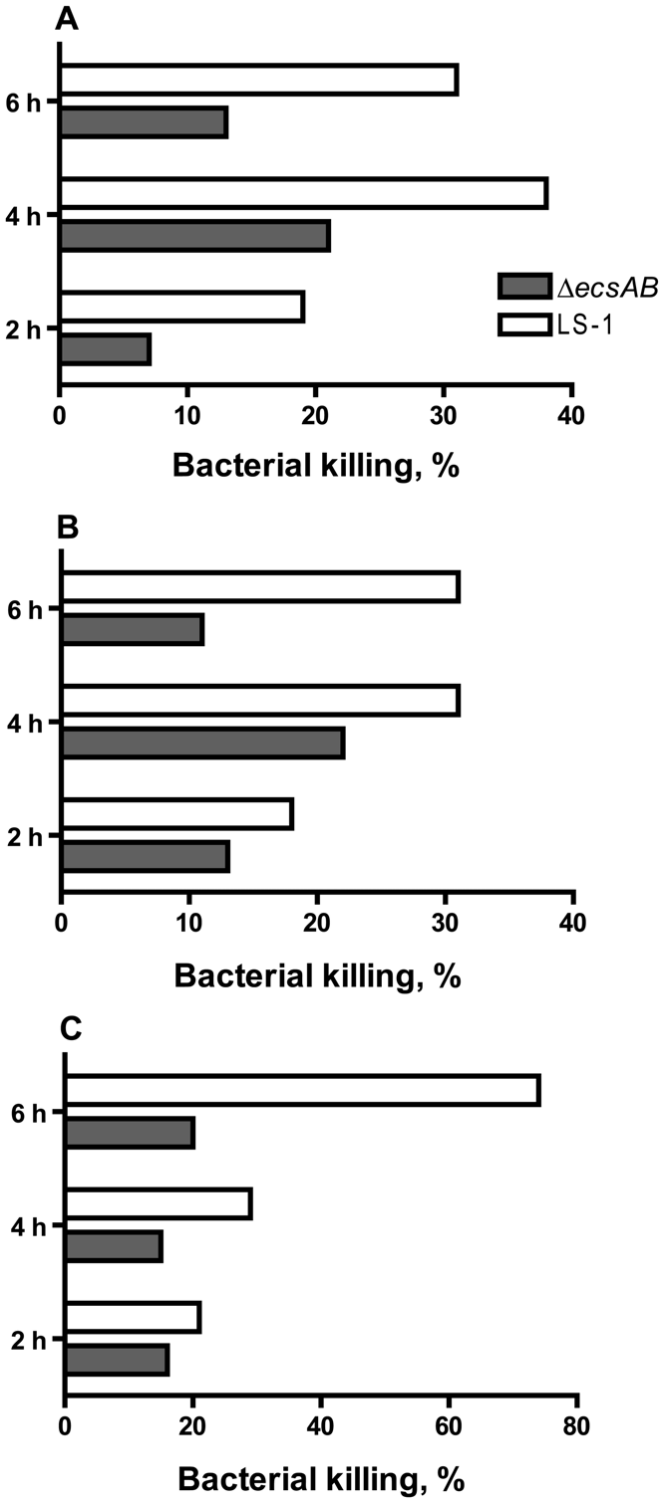
Bactericidal effect of α-defensin HNP-2 on *S. aureus* LS-1 and its isogenic Δ*ecsAB* mutant. LS-1 and Δ*ecsAB* mutant were suspended in Todd-Hewitt broth in concentrations 10^3^ cfu/ml (A), 10^4^ cfu/ml (B), and 10^5^ cfu/ml (C), and incubated with shaking in the presence of HNP-2 (5 µg ml^−1^) at 37°C. The results of 4 independent experiments are summarized. Staphylococcal growth in the presence and absence of HNP-2 was evaluated at determined time points and given in percent, indicating bacterial killing effect of HNP-2. In the tested bacterial concentrations, the bactericidal effect of HNP-2 was 2–4 times stronger on LS-1 as compared to Δ*ecsAB* mutant.

We investigated if the decreased virulence of the *ecs* mutants could be explained by increased sensitivity to host antibacterial defensins. The bactericidal effect of α-defensin HNP-2 was evaluated using wild-type LS-1 and Δ*ecsAB* mutant strains. HNP-2 at concentrations 1–5–10 µg/ml showed a dose and time dependent bactericidal effect on both wild-type and Δ*ecsAB* strains. The Δ*ecsAB* mutant was less sensitive to the bactericidal effect of HNP-2 at all concentrations of staphylococci (Figure 5). In the presence of 5 µg/ml of HNP-2, the bacterial counts of Δ*ecsAB* mutant were 2–4 times higher as compared to wild-type LS-1 strain.

### Increased susceptibility of the *ecsA*::intron mutant to ribosomal antibiotics and chelerythrine and sanguinarine plant alkaloids

To explore further potential phenotypes of *ecs* mutant, a phenotype microarray analysis (Biolog) was carried out with 20 different 90-well microtiter plates that tested susceptibility of the mutant (*ecsA*::intron) to numerous antimicrobial agents and stress conditions, and the ability to grow on various nutrients. In Biolog phenotype microarrays, reduction of the tetrazolium indicator dye is measured as a response to general metabolic activity of cells. This kind of phenotype screen may reveal molecules that affect the same cellular functions as Ecs and result in identification of new lead molecules for antimicrobial development.

The analysis suggested that *ecsA*::intron mutation increased the susceptibility to several antimicrobial agents (Figure S2). The *ecsA*::intron mutant appeared to be more sensitive to chloramphenicol, sisomicin, tobramycin and paromomycin, which are ribosomal antibiotics that inhibit protein synthesis, than the wild-type strain. The mutation also increased the susceptibility to antimicrobial agents other than antibiotics. Two plant alkaloids, chelerythrine and sanguiranine, and some drugs and chemicals with antimicrobial activity, including atropine, amitriptyline, ethylene glycol, lipoic acid, orphenatrine, phenylarsine oxide, DL-propranolol and thioridazine, inhibited the *ecs* mutant at lower concentrations than the wild-type strain. A minor defect was also observed in the mutant's ability to utilize glutamate/glutamine-containing dipeptides as a sole nitrogen source.

To further examine the Biolog findings we determined the minimal inhibitory concentrations (MICs) of a number of the Biolog-identified antimicrobial agents in Bioscreen growth experiments. The MIC determinations were performed by cultivating the two strains in BHI medium on Bioscreen plates with additives and measuring optical densities, *i.e.* growth, of the cultures (see Materials and Methods for details). Consistently with the phenotype microarray data, *ecsA*::intron clearly decreased MICs of the ribosomal antibiotics tobramycin, sisomycin and chloramphenicol (Table 3). Susceptibility of the mutant to chelerythrine and sanguinarine was also increased (about 2-fold difference in MICs), while no significant differences were observed in the sensitivity to amitriptyline, chlorpromazine, lipoic acid, phenylarsine oxide, DL-propranolol or thioridazine.

**Table 3:** Minimal inhibitory concentrations of selected antimicrobial agents.

Antimicrobial agent	MIC (µg ml^−1^)	
	*S. aureus* Newman	*S. aureus ecsA*::intron
Phenylarsine oxide	7.5	5
Chelerythrine chloride	12.5	5
DL-Propranolol hydrochloride	400	350
Chlorpromazine hydrochloride	50	50
Lipoic acid	2000	1750
Amitriptyline hydrochloride	150	125
Thioridazine hydrochloride	20	15
Sanguinarine chloride hydrate	10	5
Chloramphenicol	4	2
Tobramycin	7.5	2.5
Sisomicin	5	1

Numbers are concentrations resulting in no growth in 16 h experiment. Three replicates, each experiment repeated at least two times.

## Discussion

In this study we mutated the *ecsAB* genes of *S. aureus* and characterized the changes in transcriptome profile, phenotype and functional properties of staphylococci in relation to this mutation including *in vivo* evaluation of virulence. We show that the EcsAB transporter is dispensable for growth but critical for virulence and thus may be a potential novel antimicrobial drug target.

Results obtained in the mouse model of hematogenous staphylococcal infection show that the Ecs deficiency attenuates all signs of infection including weight loss, persistence of bacteria in kidneys, frequency and severity of arthritis and bone destruction, and finally results in a significant reduction in mortality. The identical findings obtained in the experiments using two different types of *ecs* mutations and two host strains strongly imply that the observed attenuated virulence was caused by the absence of Ecs. Another way of confirming this would be to use a complemented mutant strain. However, due to known difficulties in controlling plasmid maintenance during *in vivo* infection this option was not used in our study.

We evaluated the mechanisms leading to the decreased virulence of the *ecs* mutants. The expression profile of transporter and adhesion proteins revealed significant changes in the *ecsA*::intron mutant as compared to the wild-type strain. The expression of the *spa* gene was strongly repressed (about 20 fold) in the *ecsA*::intron mutant and the *spa* repression resulted in decreased Protein A levels in the mutant. Protein A, mediating resistance to opsonization and phagocytosis, is a known virulence factor in *S. aureus* arthritis and sepsis [21] as well as in subcutaneous experimental infections in mice [22]. Protein A is also involved in *S. aureus* invasion of cultured epithelial cells [23] and development of biofilm-associated infections [24]. The *sdrC* and *sdrD* genes encoding virulence-related adhesins were down-regulated in the *ecsA*::intron mutant. The expression of these genes is positively regulated by the virulence factor regulator protein Rot [25], [26]. The *rot* gene is down-regulated in the *ecsA*::intron mutant (2.7–3.5 fold) which may cause the down-regulation of adhesins in the *ecs* mutant. The inability of the *ecsA*::intron mutant to increase cellular level of the IsdA iron-regulated heme-iron binding protein inder conditions in which iron availability is limited may lead to impaired growth and inability to cause severe infections. In the absence of Ecs, significant changes occur in the cell wall, as evidenced by increased autolysis and sensitivity to lysostaphin, altered cell surface texture as well as decreased levels of cell wall proteins. All these phenotypes are probably consequences of the main transport defect in the membrane and may be reasons for the low virulence of *ecs* mutants.

In a previous study, signal peptides of overexpressed secretory proteins were found to be processed inefficiently in *ecs* mutants of *B. subtilis* and increased levels of preAmyQ α-amylase precursor was detected on the outer surface of the cell membrane, suggesting a defect in a late stage of protein secretion [8]. We did not observe a similar impaired signal peptide processing in the *S. aureus ecsA*::intron mutant, rather an effect on the release from the membrane and accumulation in the cell wall of preSak-Myc. The level of preSak-Myc was low in the wall of the mutant, in contrast to its abundant amount in the wall of the wild-type strain, which is consistent with transport disability. The Myc-tagged staphylokinase was expressed from the xylose-inducible T5X promoter, and its levels were similar in the whole cells and in protoplasts of the two strains (induction with 0.5% xylose). Thus, the difference in the accumulation of preSak-Myc in the wall of *ecsA*::intron mutant and wild-type strains is unlikely to be induced at the transcription level.

The *B. subtilis* σ^W^ anti-sigma factor RsiW and the FtsL cell division protein are membrane proteins with single transmembrane segments which are processed inside the lipid bilayer of the membrane by the RasP (YluC) intramembrane-cleaving protease [11], [27], [28]. The processing of RsiW and FtsL is inhibited in *ecs* mutants of *B. subtilis* [11]. One possible explanation for this inhibition is that membrane-spanning peptides or proteins accumulate in the membrane and competitively inhibit RasP. It can be speculated that Ecs is involved in the removal and cleaning of such peptides/proteins from the membrane. The finding that the release of overexpressed preSak-Myc was decreased in the *S. aureus ecsA*:.intron mutant as compared to the wild-type strain suggests this kind of transport function for Ecs. In future studies the transport function must be confirmed in a reconstituted system with purified EcsAB proteins and peptide and preSak-Myc substrates.

The inactivation of Ecs increased susceptibility of *S. aureus* to several antimicrobial agents including ribosomal antibiotics as was demonstrated by phenotype microarray analysis and MIC determinations. The increased susceptibility may be related to the altered expression of several genes encoding ribosomal proteins observed in the DNA microarray. Interestingly, the *ecs* mutant was also more sensitive to chelerythrine and sanguinarine plant alkaloids. These alkaloids are antimicrobial compounds, but their mode of antimicrobial action is unclear. We showed that Δ*ecsAB* mutation decreased susceptibility to HNP-2 α-defensin. α-Defensins are antimicrobial peptides secreted by polymorphonuclear cells and involved in the first line of defense against invading microbes [29]. HNP-2 is lethal to bacteria since it forms pores in the cytoplasmic membrane [30], [31]. This observation is unexpected taking into consideration the increased sensitivity of the *ecsA*::intron mutant to autolysis, lysostaphin as well as to antimicrobial agents. The mechanism of such a discrepancy needs further evaluation.

It is still a mystery which molecule(s), if any, Ecs transports. Since obligatory anaerobes or microaerophilic bacteria do not have Ecs, the function of Ecs might be related to respiration. We have performed experiments to find out whether Ecs transports heme to cytochromes in *B. subtilis*. Cytochrome C-bound heme in a null mutant of *B. subtilis ecsAB* and its wild-type parent was labeled with 5-[4-^14^C]aminolevulinic acid and analyzed in SDS-PAGE as has been described [32]. The result showed that Ecs is not involved in heme transport (unpublished). In this study, we showed with *S. aureus* that Ecs does not affect the proton motive force either.

Our study shows that the Ecs deficiency attenuates staphylococcal virulence and increases its sensitivity to antimicrobial agents. This is probably a consequence of the defects in the composition and function of the bacterial membrane and cell wall.

## Materials and Methods

### Ethics statement

Ethical permission (282-06) was obtained from the Animal ethical committee of Göteborg, Sweden.

### Bacterial strains and culture conditions

*S. aureus* strains used in this study are Newman [15], LS-1 [14] and RN4220 (NCTC 8325-4 r-m+). *Escherichia coli* DH5α was used for cloning. Staphylococci were grown in BHI (Brain Hearth Infusion) medium and on corresponding agar plates if not otherwise indicated. Iron-depleted BHI medium was prepared by treating BHI with 5% Chelex 100 for 1 hour. *E. coli* was cultivated in LB (Luria-Bertani) medium and on corresponding agar plates. Growth media were supplemented with chloramphenicol (10 µg ml^−1^), erythromycin (2.5 µg ml^−1^) or ampicillin (100 µg ml^−1^) when needed to maintain plasmids in the strains.

### Null mutations of *ecsAB*

In order to construct the Δ*ecsAB* mutation, the plasmid pKTH3726 was constructed. The *ecs*-deletion cassette in pKTH3726 was constructed by PCR-amplifying two approximately 1 kb fragments, ecs-up and ecs-down, and fusing them by ligating the *Sph*I site in the 3’ end of ecs-up to the *Sph*I site in the 5’ end of ecs-down. The primers used to amplify ecs-up were: ecs-1, 5’ GGGGACCACTTTGTACAAGAAAGCTGGGTTTGSAAACTACCAAATTCCGCAC and ecs-2, 5’ GGAAGCATGCCAGGGCGTTTTTCCATATCCAC. The primers used to amplify ecs-down were: ecs-3, 5’ GGAAGCATGCTCTCAATTTATGCGTCATGCG and ecs-4, 5’ GGGGACAAGTTTGTACAAAAAAGCAGGCTCATTACTTTGGTTCAAGCGGAC. pKTH3726 was obtained by inserting the *ecs*-deletion cassette between the attB sites in the pKOR1 plasmid by using Clonase (Invitrogen) as previously described [33]. The *ecsAB* operon (SA1655 and SA1654, respectively) in the *S. aureus* strain LS-1 was disrupted by using pKTH3726 and the procedure described previously [33], [34]. The Δ*ecsAB* mutation deleted the fragment from the nucleotide +50 of *ecsA* (the first nucleotide of the coding region is +1) to +285 of *ecsB*.

The *ecsA*::intron mutation was constructed by using the TargeTron system (Sigma-Aldrich) with the vector plasmid pNL9164 according to the instructions of the manufacturer. For the re-targeting of the intron II RNA into *ecsA,* a 350 bp re-targeting DNA fragment was amplified in a one step assembly PCR and inserted into pNL9164. The primers used in the PCR were: 309/310s-IBS; 5’ AAAAAAGCTTATAATTATCCTTAGCAATCGCATATGTGCGCCCAGATAGGGTG, 309/310s-EBS1d; 5’ CAGATTGTACAAATGTGGTGATAACAGATAAGTCGCATATGATAACTTACCTTTCTTTGT, 309/310-EBS2; 5’ TGAACGCAAGTTTCTAATTTCGATTATTGCTCGATAGAGGAAAGTGTCT and EBS Universal; 5’ CGAAATTAGAAACTTGCGTTCAGTAAAC.

### Construction of plasmids for expression of EcsAB and Myc-tagged staphylokinase

The pKTH3832 plasmid was constructed by PCR-amplifying a fragment containing the ribosome-binding site and coding regions of the *ecsAB* operon with the primers SaecsAB-fw and SaecsAB-rv and inserting the fragment into the pEPSA5 plasmid [16] between the *Sac*I and *Bam*HI sites. The primer sequences are: SaecsAB-fw; 5’ CACAGAGCTCCAATATAAGTCATGGAGGTGCCTTATG and SaecsAB-rv; 5’ CACAGGATCCTTAGTCTCGTAATAATGTTTCCTGATATTTCA.

The pKTH3834 plasmid was constructed in a similar manner as pKTH3832 but the *sak*-*myc* fragment was inserted between the *Eco*RI and *Bam*HI sites in pEPSA5. The *sak*-*myc* fragment was PCR-amplified with the primers sak-fw; 5’ CACAGAATTCTTATATTTGGAGGAAGCGCCAT and sak-rv; 5’ CACAGGATCCTTACAGATCTTCTTCGCTGATCAGTTTCTGTTCTTTCTTTTCTATAATAACCTTTGTAATTAAGTTGAATCC.

### Growth tests and susceptibility to antimicrobial agents

Growth of bacteria and determination of minimal inhibitory concentrations (MICs) of different antimicrobial agents were performed using a Bioscreen C apparatus (Growth Curves, Helsinki, Finland). For starting inocula, 5 ml of BHI medium was inoculated with single colonies and grown for at least 20 h at 37°C with continuous shaking. Overnight cultures were then diluted 1∶30 in BHI medium and growth was monitored with Klett Summerson colorimeter until early exponential growth phase. Cultures were diluted 100 fold with BHI and a matrix of wells with dilution series of the antimicrobial compounds in BHI were set up on 100-well honeycomb plates (Growth Curves, Helsinki, Finland). The inoculum was 15 µl and final volume 150 µl per well. Plates were incubated for 16 h at 37°C with moderate continuous shaking and growth was measured with a wide-band filter at 15 min intervals.

### Autolysis and lysostaphin lysis assays

Autolysis of *S. aureus* cells was essentially tested as originally described by de Jonge and collaborators [35], but lysis was monitored with a Bioscreen apparatus. Inocula were grown as described above for the growth experiments. Cultures were grown to mid exponential or stationary growth phases and then harvested, washed and resuspended in autolysis buffer. Samples were diluted to an initial optical density (as measured in the Bioscreen apparatus) of about 0.5 and volume of 150 µl. Decay of cells was monitored as described above for growth with continuous low level shaking.

Lysostaphin sensitivity was assayed as has been described previously [36]. After adding 10 µg ml^−1^ final concentration of lysostaphin, bacterial lysis was monitored with the Bioscreen by reading OD_600_ values at 2 min intervals.

### Microscopy

Electron microscopy samples were prepared from exponential phase cultures by adding 2.1% final concentration of glutaraldehyde to 5 ml of culture in a Parafilm M foil-secured 14 ml Falcon polypropylene test tube (Becton Dickinson). To enhance fixation tubes were slightly pressurized by injecting them with 1 ml of air through the sealed cap using a small needle, followed by incubation overnight at 20–22°C. The fixed cells were washed twice in phosphate buffer and then treated with 1% osmium tetroxide for 1 h. The samples were dehydrated with a series of treatments with ethanol and acetone, followed by embedding in Epon resin. The transmission and scanning electron microscopy was performed in the Institute of Biotechnology, University of Helsinki, by using JEOL 1200EX II and Zeiss DSM-962 electron microscopes, respectively.

### Immunoblot analysis of cell wall proteins and preSak-Myc

Protoplast and cell wall subcellular fractions were isolated by harvesting cells from 1 ml of culture and incubating the cells in 0.1 ml of the protoplast buffer (20 mM potassium phosphate pH 7.5, 15 mM MgCl_2_ and 20% sucrose) containing 0.1 mg/ml lysostaphin for 30 min at 37°C, followed by centrifugation at 5,000 rpm in an Eppendorf miniSpin Plus centrifuge for 10 min. The protoplast pellet was resuspended in 0.1 ml of protoplast buffer, followed by adding 25 µl of 5×Laemmli sample buffer to the protoplast fraction as well as the protoplast supernatant (cell wall fraction) and heating for 10 min at 100°C. Whole cell fractions were prepared in a similar manner but the centrifugation step was omitted. Proteins in 10 µl samples were separated in SDS-PAGE and analyzed by immunoblotting with specific antibodies. Cell wall proteins were detected with rabbit antibodies and Protein A-peroxidase (Sigma-Aldrich). Myc-tagged staphylokinase was detected with HRP-conjugated mouse anti-Myc antibodies (ab62928) from Abcam. ECL reactions were performed using an Immun-Star WesternC kit (Bio-Rad) and proteins were visualized and quantitated with a FluorChem HD2 imager and AlphaEase FC software (AlphaInnotech).

### Mouse model of *S. aureus* arthritis

Six to eight weeks old female NMRI mice (Scandbur, Uppsala, Sweden or from Charles River Laboratories, Sullzfeld, Germany) were used. Mice were maintained in the animal facility of the Department of Rheumatology and Inflammation Research, University of Gothenburg, Sweden, housed under standard conditions of temperature and light, and fed standard laboratory chow and water ad libitum. Ethical permission was obtained from the Animal ethical committee of Göteborg, Sweden.

Mice were inoculated intravenously into the tail vein with 6.4–7×10^6^ colony forming units (CFU) of the wild-type LS-1 strain (n = 35) or 6.6–9×10^6^ CFU of the Δ*ecsAB* mutant strain (n = 35) in a total volume of 200 µl of phosphate-buffered saline (PBS). Before inoculation, aliquots of bacteria frozen in PBS containing 5% bovine serum albumin w/v (BSA) and 10% v/v dimethyl sulfoxide (DMSO) after over night culture on horse blood agar plates overnight followed by transfer to fresh agar plates and incubation for an additional 24 h period were thawed, washed, diluted in PBS, and adjusted to the appropriate concentration. Viable counts were performed to determine the number of bacteria injected in each experiment.

Mice were also inoculated as described above with 5.9×10^6^ CFU of the wild-type Newman strain (n = 10) or 6.6×10^6^ CFU of the isogenic *ecsA*::intron mutant strain (n = 10).

Mortality was recorded and all mice were weighted and graded blindly by one observer (I-M J) for arthritis severity and frequency at regular intervals. Finger/toe and wrist/ankle joints were inspected visually for signs of arthritis defined as erythema and or swelling. Arthritis was scored from 0 to 3 points for each limb (0 = normal appearance; 1 = mild swelling and/or erythema; 2 = moderate swelling and erythema; 3 = marked swelling and erythema).

Blood, limbs and kidneys were taken at day 3, 14 and 17 from the Δ*ecsAB* mutant and LS-1 strain inoculated mice.

Kidneys were taken at day 14 from *ecsA*::intron mutant strain and Newman inoculated mice.

### Histologic examination of joints

Histopathological evaluation of the joints was done after routine paraformaldehyde fixation, decalcification, and paraffin embedding. Tissue sections from fore- and hind-paws were stained with hematoxylin and eosin and microscopically scored with regard to synovial hypertrophy/infiltration of leukocytes and cartilage and bone erosion, by an observer (I-M J) blinded to inoculation groups. Synovitis and erosion of cartilage and/or bone was scored; 0 = normal appearance, 1 = mild synovitis and/or erosion of cartilage and bone, 2 = moderate synovitis and erosion of cartilage and/or bone, 3 =  severe synovitis and erosion of cartilage and bone.

### Bacteriological examination of infected animals

The kidneys were aseptically removed, homogenized on ice, serially diluted in PBS and spread on horse blood agar plates. The number of CFU per kidney pair was determined after incubation for 24 h at 37°C.

### Serological analysis of IL-6 levels

The serum levels of interleukin-6 was determined using the murine hybridoma cell line (B9) as previously described [37].

### Statistical analyses of the mouse infections

Statistical analyses were performed by using the GraphPad Prism (LaJolla, CA). Statistical differences between independent groups were calculated using Mann-Whitney U test or Fischer's exact probability test. Kaplan-Meier survival plots were prepared and the log-rank test was used for comparison between survival curves. P-values <0.05 were considered as statistically significant.

### Influence of α-defensins on growth of the isogenic *S. aureus* strains

LS-1 and its isogenic Δ*ecsAB* mutant were suspended in Todd-Hewitt broth (THB) in concentrations 10^3^, 10^4^, and 10^5^ cfu/ml, and incubated shaking with human neutrophil peptide-2 (HNP-2, Bachem, Finechemikalien AG, Switzerland) at a range of 0–10 µg ml^−1^ at 37°C. At determined time points, 0.1 ml of the cultures were spread on blood agar plates and incubated over night at 37°C. The number of staphylococcal colonies were counted and compared between LS-1 and Δ*ecsAB* strains. To assess the effect of HNP-2 on staphylococcal growth, reduction in cfu count of HNP-2-treated cultures was compared to cfu count of untreated cultures. The results are presented as percent of bacterial killing.

### Phenotype microarray (PM) analysis

The PM analysis was carried out by using the service of Biolog (Hayward, CA). The method has been described in other studies [34], [38].

### Preparation of labeled nucleic acids for expression microarrays

Total RNA was purified from bacteria grown in BHI for 3 or 6 h. The inocula were 50-fold dilutions from overnight cultures. For each strain, RNA of four independently grown cultures was analyzed. After an additional DNase treatment, the absence of remaining DNA traces was confirmed by quantitative PCR (SDS 7700; Applied Biosystems, Framingham, MA) with assays specific for 16S rRNA [39]. Batches of 5 µg of total *S. aureus* RNA were labeled with Cy3-dCTP (RH7783) or Cy5-dCTP (RH7603) using SuperScript II (Invitrogen, Basel, Switzerland) following manufacturer's instructions. Labeled products were then purified on QiaQuick columns (Qiagen). A mixture of Cy-3 and Cy-5 labeled products (250 ng) were mixed for each time point, diluted in 60 µl Agilent hybridization buffer, and hybridized at a temperature of 60°C for 17 hours in a dedicated hybridization oven (Robbins Scientific, Sunnyvale, CA). Slides were washed with Agilent proprietary buffers, dried under nitrogen flow, and scanned using 100% PMT power for both wavelengths.

### Microarray analysis

Fluorescence intensities were extracted using Feature Extraction software (version 8; Agilent). Local background-subtracted signals were corrected for unequal dye incorporation or unequal load of the labeled product. The algorithm consisted of a rank consistency filter and a curve fit using the default LOWESS (locally weighted linear regression) method. Data consisting of two independent biological experiments were expressed as log 10 ratios and analyzed using GeneSpring, version 8.0 (Silicon Genetics, Redwood City, CA). Statistical significance of differentially expressed genes was calculated by analysis of variance [40] using GeneSpring, including the Benjamini and Hochberg false discovery rate correction of 5% (*P* value cutoff, 0.05) and an arbitrary cutoff of twofold for expression ratios.

### Microarray data accession number

Microarray design has been described previously [39]. The complete microarray data set has been posted on the Gene Expression Omnibus database (http://www.ncbi.nlm.nih.gov/geo/) under accession number GPL10537 for the platform design and GSE22353 for the original data set.

## Supporting Information

Figure S1 Two-way clustering analysis of *S. aureus* transcriptomic patterns. Expression microarrays (normalized data) were clustered by a hierarchical clustering algorithm by using an average linkage method in GeneSpring. The expression values for a gene across all samples were standardized to have mean of 0 and standard deviation of 1 by linear transformation. To determine the amount of detectable genes, the expression values were averaged for transcripts mapped by 2 or more probes. A cut-off value defined as 2 x standard deviation obtained for background intensities was then applied.The figure shows a clear separation of 4 sub-clusters containing 4 replicate experiments (1-4) for each time-point and for the WT (RH7603) or the mutant strain (RH7783). Each probe set is represented by a single row of colored boxes and each sample corresponds to a single column. The blue areas correspond to genes showing high or medium expression whereas yellow bars indicates genes poorly or not expressed. The dendrogram (green lines) on the top of the figure represents the similarity matrix of probe sets.
(3.51 MB DOC)

Figure S2 Phenotype microarray analysis of the *ecsA*::intron mutant. Antimicrobial sensitivities of RH7783 (*ecsA*::intron) and RH7603 (*S. aureus* Newman) were compared by using phenotype microarrays. Scatter plots of parameter values from two replicates of the PM analysis are shown in the two uppermost panels. Three other panels show overlaid color-coded images of tetrazolium reduction kinetics (mutant versus wild type) over all wells in the two runs of the analysis and their consensus. Phenotypes observed are listed below the figure panels.
(5.70 MB RTF)

Table S1 Genes induced or repressed in cells of the *ecsA*::intron mutant in the late exponential growth phase (3h) as compared to the wild-type Newman strain.
(0.07 MB XLS)

Table S2 Genes induced or repressed in cells of the *ecsA*::intron mutant in the early stationary growth phase (6h) as compared to the wild-type Newman strain.
(0.08 MB XLS)

Table S3 Persistence of *ecs* mutant and wild-type *S. aureus* cells in kidneys.
(0.04 MB RTF)
