# Supplementary material for: Inactivation of the Ecs ABC Transporter of Staphylococcus aureus Attenuates Virulence by Altering Composition and Function of Bacterial Wall
Source: PLoS One. 2010 Dec 2;5(12):e14209. doi: 10.1371/journal.pone.0014209 (PMC2996298; doi:10.1371/journal.pone.0014209)
Supplement: Figure S1 — Two-way clustering analysis of S. aureus transcriptomic patterns. Expression microarrays (normalized data) were clustered by a hierarchical clustering algorithm by using an average linkage method in GeneSpring. The expression values for a gene across all samples were standardized to have mean of 0 and standard deviation of 1 by linear transformation. To determine the amount of detectable genes, the expression values were averaged for transcripts mapped by 2 or more probes. A cut-off value defined as 2 x standard deviation obtained for background intensities was then applied.The figure shows a clear separation of 4 sub-clusters containing 4 replicate experiments (1-4) for each time-point and for the WT (RH7603) or the mutant strain (RH7783). Each probe set is represented by a single row of colored boxes and each sample corresponds to a single column. The blue areas correspond to genes showing high or medium expression whereas yellow bars indicates genes poorly or not expressed. The dendrogram (green lines) on the top of the figure represents the similarity matrix of probe sets. (3.51 MB DOC) [file pone.0014209.s001.doc]

**Figure S1**: Two-way clustering analysis of *S. aureus* transcriptomic patterns. Expression microarrays (normalized data) were clustered by a hierarchical clustering algorithm by using an average linkage method in GeneSpring. The expression values for a gene across all samples were standardized to have mean of 0 and standard deviation of 1 by linear transformation. To determine the amount of detectable genes, the expression values were averaged for transcripts mapped by 2 or more probes. A cut-off value defined as 2×standard deviation obtained for background intensities was then applied (Scherl, 2005).The figure shows a clear separation of 4 sub-clusters containing 4 replicate experiments (1-4) for each time-point and for the WT (RH7603) or the mutant strain (RH7783). Each probe set is represented by a single row of colored boxes and each sample correspond to a single column. The blue areas correspond to genes showing high or medium expression whereas yellow bars indicates genes poorly or not expressed. The dendrogram (green lines) on the top of the figure represents the similarity matrix of probe sets.

Scherl A, Francois P, Bento M, Deshusses JM, Charbonnier Y, Converset V, Huyghe A, Walter N, Hoogland C, Apel RD, Sanchez JC, Zimmerman-Ivol CG, Corthals GL, Hochstrasser DF, Schrenzel J. Journal Microbiological Methods 2005; 60(2):247-257.
