# Supplementary material for: Inactivation of the Ecs ABC Transporter of Staphylococcus aureus Attenuates Virulence by Altering Composition and Function of Bacterial Wall
Source: PLoS One. 2010 Dec 2;5(12):e14209. doi: 10.1371/journal.pone.0014209 (PMC2996298; doi:10.1371/journal.pone.0014209)
Supplement: Figure S2 — Phenotype microarray analysis of the ecsA::intron mutant. Antimicrobial sensitivities of RH7783 (ecsA::intron) and RH7603 (S. aureus Newman) were compared by using phenotype microarrays. Scatter plots of parameter values from two replicates of the PM analysis are shown in the two uppermost panels. Three other panels show overlaid color-coded images of tetrazolium reduction kinetics (mutant versus wild type) over all wells in the two runs of the analysis and their consensus. Phenotypes observed are listed below the figure panels. (5.70 MB RTF) [file pone.0014209.s002.rtf]

FIG. S2. Antimicrobial sensitivities of RH7783 (ecsA::intron) and RH7603 (S. aureus Newman) were compared by using phenotype microarrays. Scatter plots of parameter values from two replicates of the PM analysis are shown in the two uppermost panels. Three other panels show overlaid color-coded images of tetrazolium reduction kinetics (mutant versus wild type) over all wells in the two runs of the analysis and their consensus. Phenotypes observed are listed below the figure panels.


Replicate 1 versus Replicate 2 of Test:
Scer_SID_5648_RH7783


Replicate 1 versus Replicate 2 of Reference:
Saur_SID_5645_RH7603


Run 1:
Scer_SID_5648_RH7783( green )
 versus
Saur_SID_5645_RH7603( red )


Run 2:
Scer_SID_5648_RH7783( green )
 versus
Saur_SID_5645_RH7603( red )


Consensus:
Scer_SID_5648_RH7783( green )
 versus
Saur_SID_5645_RH7603( red )


PM Report:
Scer_SID_5648_RH7783_0.002% YE, Dye D
 versus 
Saur_SID_5645_RH7603_0.002% YE, Dye D

Phenotypes Gained: None

Phenotypes Lost:
PM20B	C08	-169	Atropine		acetylcholine receptor, antagonist
PM17A	B02,B03,B04	-297	Salicylate		anti-capsule, anti-inflammatory, mar inducer
PM14A	A09	-168	Sanguinarine		ATPase, Na+/K+ and Mg++
PM20B	B08	-151	D,L-Propranolol		beta-adrenergic blocker
PM15B	C11	 -97	1,10-Phenanthroline		chelator, Fe++, Zn++, divalent metal ions
PM15B	B09	 -62	5,7-Dichloro-8-hydroxy-quinaldine	chelator, lipophilic
PM20B	B03	-171	Orphenadrine		cholinergic antagonist
PM14A	H07	 -98	Promethazine		cyclic nucleotide phosphodiesterase
PM18C	H08	 -60	2- Phenylphenol		DNA intercalator
PM20B	G01	 -62	Captan		fungicide, carbamate, multisite
PM18C	D09	 -96	Lidocaine		ion channal inhibitor, Na+ 
PM15B	A05	-161	Guanidine hydrochloride		membrane, chaotropic agent
PM20B	A03	-143	Amitriptyline		membrane, transport
PM06 	D09	 -85	Glu-Gly		N-source
PM07 	F09	 -84	Trp-Glu		N-source
PM06 	B05	 -75	Arg-Ala		N-source
PM07 	D06	 -73	Pro-Gln		N-source
PM06 	B08	 -71	Arg-Gln		N-source
PM06 	C04	 -67	Arg-Trp		N-source
PM06 	D05	 -66	Gln-Gln		N-source
PM06 	D11	 -64	Glu-Trp		N-source
PM06 	H07	 -62	Leu-Glu		N-source
PM06 	C07	 -61	Asn-Glu		N-source
PM07 	G06	 -57	Tyr-Gln		N-source
PM06 	D03	 -54	Asp-Val		N-source
PM06 	F10	 -52	His-Pro		N-source
PM06 	C01	 -50	Arg-Met		N-source
PM06 	D06	 -50	Gln-Gly		N-source
PM07 	D10	 -50	Pro-Phe		N-source
PM09 	D11	-153	15% Ethylene Glycol		osmotic sensitivity, ethylene glycol
PM09 	D12	-110	20% Ethylene Glycol		osmotic sensitivity, ethylene glycol
PM09 	D10	 -78	10% Ethylene Glycol		osmotic sensitivity, ethylene glycol
PM19 	E07,E08	-173	D,L-Thioctic Acid		oxidizing agent
PM17A	D11	 -92	Chlorpromazine		phenothiazine
PM14A	G01,G02,G03	-346	Chelerythrine		protein kinase C
PM14A	F03,F04	-152	Chloramphenicol		protein synthesis
PM12B	D04	-160	Sisomicin	protein synthesis, aminoglycoside
PM12B	F04	-157	Tobramycin	protein synthesis, aminoglycoside
PM12B	C04	 -83	Paromomycin	protein synthesis, aminoglycoside
PM20B	C01	-107	Thioridazine	respiration
PM20B	D11,D12	-261	18-Crown-6-Ether	respiration, ionophore
PM17A	H09	-186	Phenylarsine Oxide	tyrosine phosphatase
PM13B	B02	 -84	Azlocillin	wall, lactam
