# Supplementary material for: Inactivation of the Ecs ABC Transporter of Staphylococcus aureus Attenuates Virulence by Altering Composition and Function of Bacterial Wall
Source: PLoS One. 2010 Dec 2;5(12):e14209. doi: 10.1371/journal.pone.0014209 (PMC2996298; doi:10.1371/journal.pone.0014209)
Supplement: Table S3 — Persistence of ecs mutant and wild-type S. aureus cells in kidneys. (0.04 MB RTF) [file pone.0014209.s005.rtf]

Table S3. Persistence of ecs mutant and wild-type S. aureus cells in kidneys.	
						
Strain and dose of inoculation	Number of mice	Day of sacrifice	CFU in kidneys	p-value	
Wild-type strain	Mutant strain	wt/mutant		Wild-type strain, Md (IQR)	Mutant strain, Md (IQR)		
LS-1, 6.4×10^6^	ecsAB, 6.6×10^6^	7/10	14	6.9×10^7^ (1.4×10^7^ to 1.2×10^8^)	0 (0 to 1.6×10^6^)	0.0012	
LS-1, 7×10^6^	ecsAB, 9×10^6^	5/5	3	1.6×10^6^ (7.4×10^5^ to 4.5×10^6^)	1.1×10^4^ (0 to 7.0×10^5^)	N.S.	
		10/10	17	1.4×10^7^ (0 to 2.8×10^7^)	0 (0 to 0)	0.0049	
Newman, 5.9×10^6^	ecsA::intron,
6.6×10^6^	5/9	14	5.4×10^7^ (3.2×10^7^ to 1.5×10^8^)	0 (0 to 500)	0.001	
